# Response to Pneumococcal Polysaccharide Vaccination in HIV-Positive Individuals on Long Term Highly Active Antiretroviral Therapy

**DOI:** 10.4172/2155-6113.1000421

**Published:** 2015-01-26

**Authors:** Anita S Iyer, David J Leggat, Jennifer A Ohtola, Joan M Duggan, Claudiu A Georgescu, Adeeb A Al Rizaiza, Sadik A Khuder, Noor M Khaskhely, Julie Westerink

**Affiliations:** 1 Department of Medicine, University of Toledo, USA; 2 Department of Medical Microbiology and Immunology, University of Toledo, USA; 3 Department of Internal Medicine, University of Toledo, USA; 4 Department of Pathology, University of Toledo, USA; 5 Department of Physiology, University of Toledo, USA; 6 Department of Pharmacology, University of Toledo, USA; 7 Department of Metabolism and Cardiovascular Science, University of Toledo, USA; 8 Department of Public Health, University of Toledo, USA

**Keywords:** HIV, HAART, *Streptococcus pneumoniae*, PPV23-vaccination, B cells, Pneumococcal-polysaccharides, Immune-response

## Abstract

**Background and objectives:**

*Streptococcus pneumoniae* continues to cause serious infections in HIV-positive individuals in the era of highly active anti-retroviral therapy. This led to the recommendation to revaccinate HIV-positive individuals with PPV23 five years after primary vaccination. The benefits of revaccination and the impact of long term highly active anti-retroviral therapy (HAART) on antigen-specific B cell reconstitution have remained unclear thus far and were investigated.

**Design and methods:**

We assessed antibody levels, opsonophagocytic activity and phenotype of pneumococcal polysaccharide (PPS) specific-B cells post-revaccination in long term HAART cohorts stratified according to CD4 count as group A (CD4>200) and group B (CD4<200). Anti-PPS IgG, IgM and functional antibody response against vaccine serotypes 14 and 23F were measured by ELISA and opsonophagocytic assay followed by phenotypic analysis of PPS14 and 23F-specific B cells using fluorescently labeled PPS.

**Results:**

Significant increases in total and functional antibody titers were noted in groups A and B post-vaccination concomitant with significant rise in PPS-specific IgM memory B cells, a critical B cell subset required for protection against PPS although the overall response remained significantly diminished compared to HIV-negative volunteers.

**Conclusion:**

Comparable increases in opsonophagocytic titers between study groups A and B concomitant with a comparable rise in PPS-specific IgM memory B cells indicate revaccination to be beneficial regardless of the degree of CD4 T cell reconstitution. These findings emphasize the importance of defining effective vaccination practices amongst high-risk individuals.

## Introduction

The advent of HAART has dramatically reduced the incidence of opportunistic-infections in HIV-positive individuals [1,2]. Despite this success, invasive pneumococcal disease (IPD) remains highly prevalent in these individuals with a disease burden 35–50 times higher than in the HIV-negative population [3,4].

High incidence of IPD in HIV-positive adults had led to the previous recommendation of revaccinating with PPV23 five years after primary immunization [5,6]. Although recent recommendations have changed to include pneumococcal conjugate vaccine (PCV13) first followed by PPV23 [7], the efficacy of PPV23 in HIV-positive individuals has thus far remained controversial. Multiple observational studies have reported little beneficial effects of PPV23 while a randomized, double blind trial in Uganda reported detrimental effects [8–14]. Previous recommendations thus stemmed from the observation that beneficial effects likely outweigh potential harm.

In addition, the ideal time for PPV23 administration and the benefits of PPV23 revaccination in HIV-positive individuals has been a subject of debate. The Center for Disease Control and Prevention (CDC) recommends pneumococcal vaccination in HIV-positive individuals with CD4 count >200 cells/µl [15]. World Health Organization (WHO) guidelines suggest that vaccination be considered in individuals with CD4 count >500 cells/µl, implying PPV23 should be administered during the early stage of HIV infection or after sufficient immune reconstitution by HAART [16]. Although reconstitution of B cells and reduction in B cell abnormalities upon HAART initiation is well documented, fewer studies have focused on the effect of HAART on antigen-specific B cell reconstitution [17].

The goal of the current study was to evaluate the impact of PPV23 revaccination in long term HAART cohorts. This is the first comprehensive study to our knowledge that discerns quantitative and qualitative anti-PPS antibody response, investigates the nature of PPS responding B cells and the influence of CD4 T cells post PPV23 revaccination in this population.

Our results show a significant rise in PPS-specific functional antibody titers in long term HAART cohorts concomitant with a rise in PPS-specific IgM memory B cells, a B cell subset critical for protection against *Streptococcus pneumoniae* [18,19] indicating PPV23 revaccination to be a beneficial practice. However, serological and PPS-specific peripheral B cell responses remained suboptimal in these individuals irrespective of the degree of CD4 T cell reconstitution as compared to our HIV-negative volunteers. These results indicate persistent PPS-specific B cell deficiencies despite long term HAART administration.

## Methods

### Study population and design

Informed consent was obtained from recruited volunteers in this University of Toledo Institutional Review Board (IRB) approved study (IRB: 106410 and 107017). HIV-positive individuals on long term HAART (≥ 5 years) were recruited from the University of Toledo Medical Center. HAART included two nucleoside analog reverse transcriptase inhibitors and one non-nucleoside reverse transcriptase inhibitor or a boosted protease inhibitor. These individuals on long term HAART had received the first dose of PPV23 ≥ 5 years ago and were eligible for PPV23 revaccination based on Advisory Committee on Immunization Practices (ACIP) recommendations at the time of enrollment [5]. They were stratified according to CD4 count at the time of vaccination as Group A: CD4>200 cells/µl (indicating immune restoration, n=29; mean age: 49) and Group B: CD4<200 cells/µl (n=10, mean age: 50). Volunteers in both groups A and B had a history of nadir CD4<200 cells/µl. Baseline characteristics of HAART cohorts are detailed in Table 1.

HIV-negative volunteers (n=22, mean age: 26) were recruited as controls and were immunized with PPV23 Merck & Co., INC (includes capsular polysaccharides from serotypes 1, 2, 3, 4, 5, 6B, 7F, 8, 9N, 9V, 10A, 11A, 12F, 14, 15B, 17F, 18C, 19F, 19A, 20, 22F, 23F, and 33F). Blood was drawn on day 0 (pre-vaccination), day 7 and 30 post-vaccination. Response to PPV23 was assessed against PPS14 and 23F for all the performed techniques. The rationale behind choosing PPS14 and 23F was based on differences in chemical structure, charge and immunogenicity [20,21]. They also served as the basis for comparison with our work in HIV-negative volunteers. All volunteers were questioned for pre-existing co-morbidities and exclusion criteria including history of cancer or leukemia, other immunosuppressing conditions, bleeding problems, pregnancy, splenectomy, organ transplant and lung disease.

### PPS-Enzyme linked immunosorbent assay (ELISA)

ELISAs were performed using day 0 and 30 volunteer serum samples along with serum standards 89SF and 007sp. Both control and volunteer serum samples were absorbed with PPS22F and cell wall polysaccharide (CWPS) based on the ELISA training manual published by World Health Organization (WHO) [22]. Briefly, Nunc Maxisorp 96 well plates were coated with 15 µg/ml PPS either 14 or 23F and incubated overnight at 37°C. Absorbed plates were washed with wash buffer (with 1X PBS, 0.05% Tween 20). After blocking the plates (1X PBS/ 1% BSA buffer) serially diluted sera were added on the plates and incubated at 37°C. Plates were washed, and bound Ab was detected using HRP-conjugated anti-human IgG or IgM (Southern Biotech). Plates were developed using o-phenylenediamine substrate and read at 490 nm on a microplate reader. Linear regression fits were used to determine the antibody concentrations and are reported as µg/ml [18].

### Opsonophagocytic assay (OPA)

Opsonophagocytic assay was performed using day 0 and 30 volunteer serum samples. Briefly, serotypes 14 and 23F were incubated with serially diluted heat-inactivated donor sera. Newborn rabbit serum (Pel-Freez, Brown Deer, WI) was added as a source of complement. Differentiated HL-60 cells were added at an effector: target (E:T) ratio of 400:1. The opsonophagocytic titer was determined as the reciprocal of the dilution with 50% killing when compared with serum free controls and analyzed using the Opsotiter1 software developed by the University of Alabama at Birmingham [18,23].

### Labeling of polysaccharides

Conjugation of PPS14 to cascade blue (CB) ethylenediamine (Invitrogen catalog C-621) or PPS23F to 5-(4,6-dichlorotriazinyl) aminofluorescein (5-DTAF; Sigma-Aldrich #36565) was carried out by Alamo Laboratories Inc, San Antonio, TX as previously described [18].

### Flow cytometry

Cells were analyzed for their ability to bind fluorescently conjugated pneumococcal PPS14 and 23F on day 0 and 7 to elucidate PPS-specific B cell phenotype as described previously [18]. Cells were stained with anti-human CD19 (APC-Cy7), CD27 (PerCP-Cy5.5), IgM (APC) to assess PPS-specific B cell subsets. A representative gating strategy is outlined in Supplementary Figure 1. Percentages of specific cell populations for each donor were determined using the outlined gating strategy. Absolute numbers of each cell populations per donor were calculated by multiplying percentage of the specific cell population with absolute lymphocyte numbers. Average values for each cell population within a group were then calculated and are reported as mean ± SEM.

### Statistical analyses

Pre- and post-vaccination data in a single group were analyzed using paired t-test. Group comparisons were performed using analysis of variance (ANOVA) with Tukey’s post-hoc test. Changes in pre- to post-vaccination response between groups were calculated by analysis of covariance (ANCOVA) with Bonferroni correction. Correlations between two groups were examined using Pearson correlation. Data is presented as mean ± standard error of the mean (SEM). p-value<0.05 were considered statistically significant. Data was analyzed using statistical analysis software (SAS).

## Results

### Serum anti-PPS antibody levels post-PPV23 revaccination in long term HAART cohorts

Anti-PPS antibody levels were evaluated pre- (day 0) and post- (day 30) vaccination.

**Figure 1:**
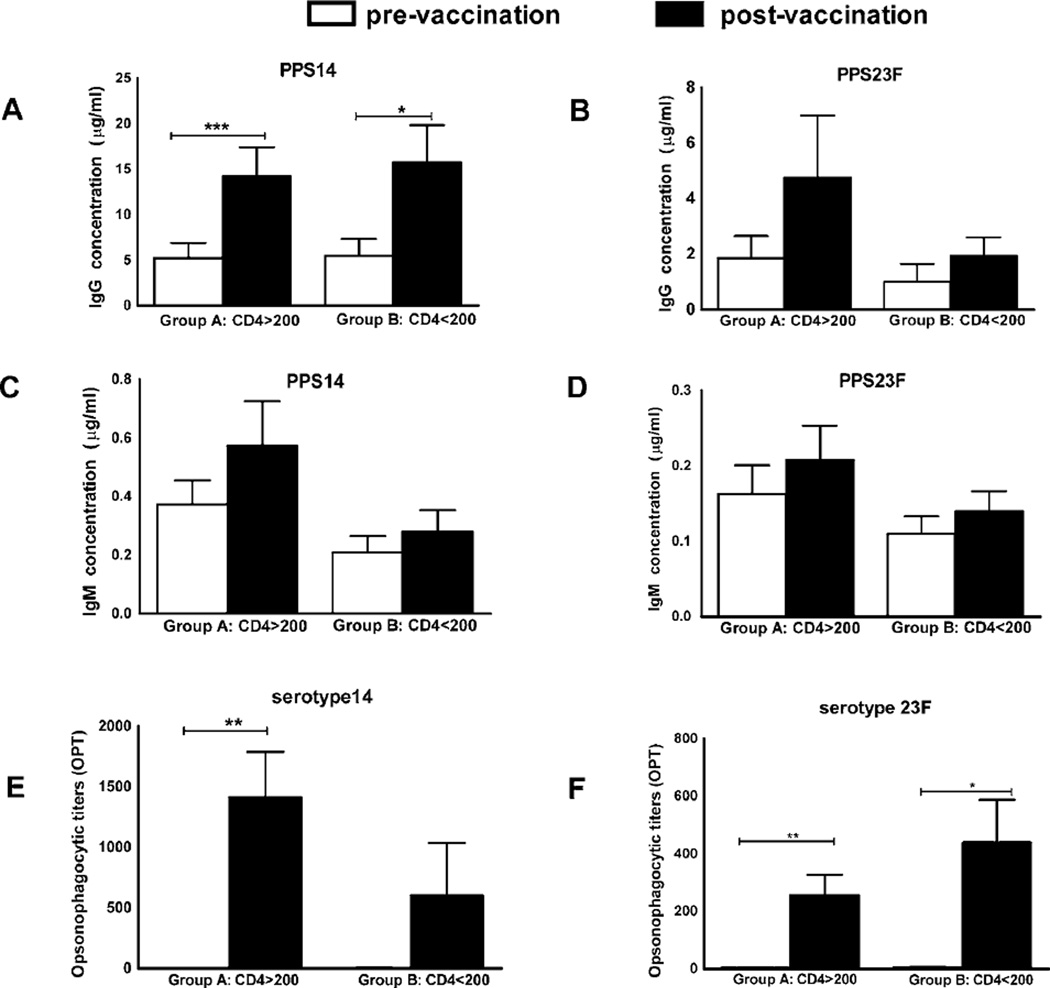
Serological response to PPV23 revaccination in long term HAART cohorts Long term HAART cohorts (group A, n=29 and group B, n=10) were immunized with PPV23. Serum samples were obtained on day 0 (pre-vaccination, white bars) and day 30 (post-vaccination, black bars) and tested for PPS14- and PPS23F-specific IgG (A, B), IgM (C, D), and opsonophagocytic activity (E, F). Serum antibody levels are expressed as µg/ml, and opsonophagocytic activity is expressed as opsonophagocytic titer (OPT). Pre to post vaccination changes in response were compared using analysis of co-variance (ANCOVA). Data are represented as mean ±standard errors of the mean (SEM). *p<0.05, **p<0.01, ***p<0.001.

Anti-PPS14 IgG response increased significantly from 5.24 ± 1.65 µg/ml to 14.23 ± 3.16 µg/ml (p ≤ 0.0001) pre-to post-vaccination in group A and from 5.48 ± 1.86 µg/ml to 15.68 ± 4.12 µg/ml (p ≤ 0.05) in group B (Figure 1A, Table 2A). Anti-PPS23F IgG response did not increase significantly in either group A or B (Figure 1B, Table 2A)

Post-vaccination, anti-PPS IgM values did not increase significantly in either group for the 2 tested serotypes. (Figure 1C, 1D, Table 2B).

Serum IgG and IgM antibodies against PPS14 and 23F levels were comparable between groups A and B, pre to post-vaccination (Figure 1A–1D and Table 2A, 2B). With the exception of anti-PPS14 IgG antibodies, overall anti-PPS specific antibody levels did not increase significantly in recruited volunteers.

### Functional antibody titers increased post PPV23 revaccination in long term HAART cohorts

Opsonophagocytic titers (OPT) are a better indication of protection since these titers indicate the cumulative killing effect of anti-PPS antibodies [24,25]. Although correlates of protection have not been established in adults, an OPT value of > 8 is used as a cutoff for pneumococcal vaccine response in studies [26,27]. Prior to vaccination, baseline OPT titers against serotype 14 was < 8 in 27 out of the 29 volunteers in group A and 8 out of the 10 volunteers in group B. Similarly, baseline OPT titers against serotype 23F OPT was < in 8 in 22 out of the 29 volunteers in group A and 7 out of the 10 volunteers in group B.

However, post-vaccination OPT increased significantly in all the recruited volunteers against the 2 serotypes and was significantly higher than the cutoff value of 8 (Table 2C). This significant rise in post-vaccination OPT was noted despite low PPS-specific antibody levels and poor correlations between IgG and IgM with OPT.

Opsonophagocytic titers (OPT) against serotype 14 increased significantly from 3.0±0.39 to 1412 ± 376 post-vaccination in group A (p≤0.001) but not in group B (Figure 1E, Table 2C).Similarly, OPT against serotype 23F increased significantly pre to post-vaccination from 5±1 to 255 ± 72 in group A (p≤0.01) and from 6±1 to 439± 148 (p≤0.05) in group B (Figure 1F, Table 2C).

Pre to post-vaccination changes in OPT were comparable between groups A and B (Figure 1 E, 1F and Table 2C).

### Absolute B cell numbers and B cell percentages in long term HAART cohorts

Absolute B cell numbers and percentages were examined in groups A and B (Table 1). We assessed 3 different populations of B cells including naïve B cells (CD27−IgM+/−), switched memory (CD27+IgM−) and IgM memory (CD27+IgM+) B cells (Table 1). No significant differences were noted between groups A and B for mentioned populations both in terms of percentage and absolute numbers.

### Phenotypic analysis of PPS-specific B cells in long term HAART cohorts pre and post-PPV23 revaccination

PPS-specific B cells peak in the peripheral blood on day 7 post-immunization [18,28]. PPS14- and 23F-specific B cell percentages and numbers increased significantly in groups A and B pre- (day 0) to post- (day 7) vaccination (Table 3).

**Figure 2:**
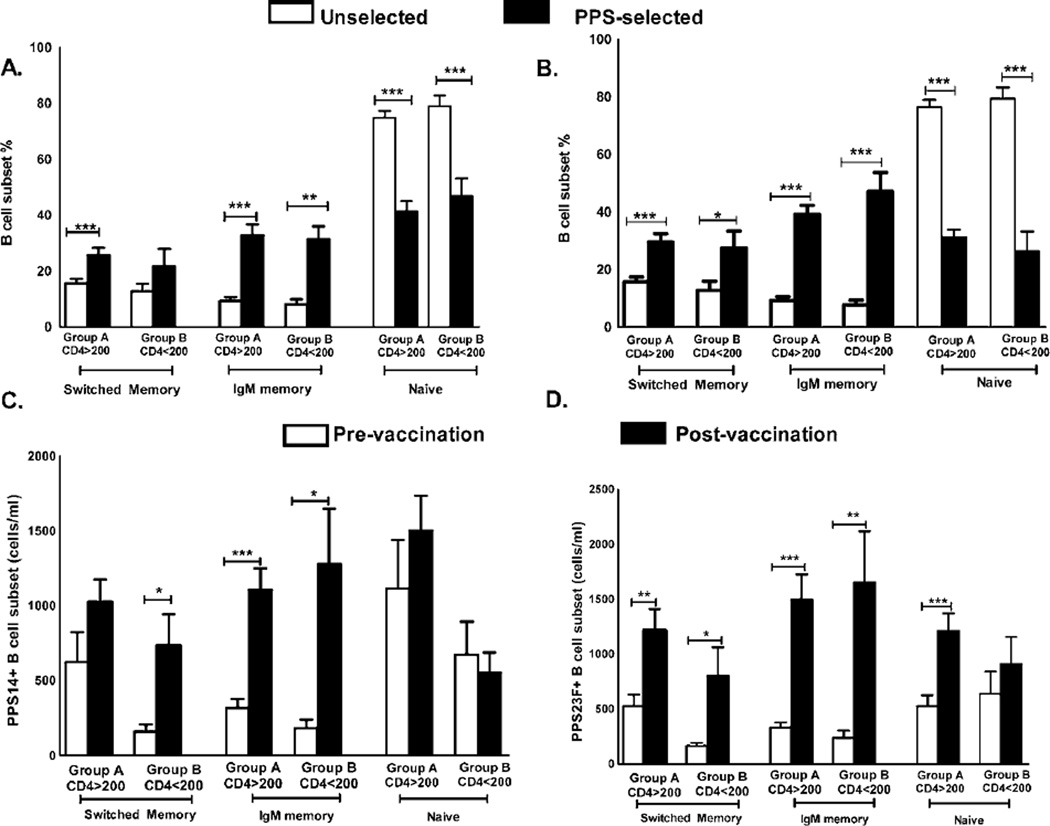
PPS-specific B cell subset response in long term HAART cohorts on day 7 post PPV23 revaccination Phenotype of B lymphocytes that respond to labeled PPS14 and 23F were determined by flow cytometry in group A (n=29) and B (n=10) post PPV immunization. *Panel A–B*: Percentage of PPS14 (A) and 23F (B) -specific B cell subsets (black bars) compared to unselected B cells (white bars). *Panel C–D*: Absolute numbers of PPS14 (C) and 23F (D) -specific switched and IgM memory B cell numbers pre (white bars) to post (black bars) vaccination. 100,000 events were recorded for each sample. Pre to post vaccination changes in response were compared using analysis of co-variance (ANCOVA).Data are represented as mean ± standard errors of the mean (SEM). *p<0.05, **p<0.01, ***p<0.001.

PPS-selected (antigen-specific) B cell subset distribution was compared to unselected B cells post-vaccination (Figure 2A and 2B, Table 4). Significant differences were noted in the B cell subset distribution between unselected and PPS-selected B cells. It should be noted that unselected B cell phenotype distribution was similar on days 0 and 7 (predominantly naive B cells, data not shown).

PPS14 and 23F-selected switched and IgM memory B cell percentages were significantly higher than the unselected counterparts in both groups A and B on day 7 (Figure 2A, 2B and Table 4A, 4B). One exception was PPS-14 specific switched memory B cell numbers in group B, comparable to unselected counterpart.

A percentage of naïve B cells (IgM^+/−^ CD27^−^) also bound to labeled PPS. However, the percentages of both PPS14 and 23F-selected naïve B cells were significantly lower than that in the unselected population in both groups A and B (Figure 2A, 2B and Table 4C).

PPS-specific B cell subset absolute numbers were assessed pre-and post- vaccination. Switched memory B cell numbers increased significantly pre- to post-vaccination in response to PPS14 in group B but not in group A (Figure 2C, Table 5A). Switched memory B cell numbers increased significantly in response to PPS23F in group A and group B (Figure 2D, Table 5A).

We also found increases in PPS14 and 23F-specific IgM memory B cell numbers in group A and B (Figure 2C, 2D Table 5B).

PPS14 and 23F-specific naïve B cells numbers did not increase significantly pre- to post-vaccination consistent with reduced percentages (Figure 2C, 2D and Table 5C). One exception was the significant increase in PPS23F-specific naïve B cells in group A (Figure 2C).

No significant differences were found in the PPS-specific B cell subset percentages and numbers between group A and B pre- to post-vaccination (Figure 2, Table 4 and 5).

We noted positive correlations between the percentages of PPS-specific but not unselected IgM memory B cells on day 7 with OPT responses on day 30. PPS14-specific IgM memory B cell percentage on day 7 correlated with anti-serotype 14 OPT on day 30 in group A (r=0.88 p <0.0001). We also noted significant correlations between the percentages of PPS23F specific IgM memory B cells in group A (r=0.61, p ≤ 0.001) and in group B (r=0.86, p ≤ 0.05) with anti-serotype 23F OPT.

Similarly, PPS14 specific IgM memory B cell absolute numbers showed positive correlations with anti-serotype 14 OPT in group A (r=0.42, p ≤ 0.05) and group B (r=0.73, p ≤ 0.05). PPS23F specific IgM memory B cell numbers correlated with anti-serotype 23F OPT (r=0.39, p ≤ 0.05) in group A but not group B (r=0.53, p=0.1).

In contrast, despite increases in the percentage and numbers of PPS14- and 23F-specific switched memory B cells in group A and B, no positive correlations were noted between these subsets and OPT.

### Diminished response to PPV23 revaccination in long term HAART cohorts as compared to HIV-negative volunteers

We compared the response to PPV23 in long term HAART cohorts to that in HIV-negative volunteers (Supplementary Figure 2). Post-vaccination anti-PPS14 IgG levels were significantly higher in HIV-negative volunteers at 35.20 ± 7.52 µg/ml than group A (14.23 ± 3.16 µg/ml, p ≤ 0.05) and group B (15.68 ± 4.1 µg/ml, p ≤ 0.05). Similarly, post- vaccination changes in anti-PPS23F IgG levels were significantly higher in HIV-negative at 13.75 ± 3.83 µg/ml volunteers than group A (4.74 ± 2.24 µg/ml, p ≤ 0.05) and group B (1.94 ± 0.66 µg/ml, p ≤ 0.05) (Supplementary Figure 2A, 2B).

Post-vaccination anti-PPS14 IgM levels were higher in HIV-negative volunteers at 12.19 ± 3.71 µg/ml compared to group A (0.57 ± 0.15 µg/ml, p ≤ 0.05) and group B (0.28 ± 0.07 µg/ml). Likewise, post-vaccination anti-PPS23F IgM values were significantly higher in HIV-negative volunteers at 8.85 ± 2.55 µg/ml compared to group A (0.20 ± 0.04 µg/ml, p ≤ 0.05) and group B (0.14 ± 0.02 µg/ml,) (Supplementary Figure 2C, D).

Consistent with ELISA results, post-vaccination OPT was significantly higher in HIV-negative volunteers compared to both group A and B (Supplementary Figure 2E, F). Serotype 14 OPT in HIV-negative volunteers was significantly higher 4395 ± 344 compared to group A (1412 ± 376, p≤0.0001) and group B (602 ± 435, p≤0.0001). Serotype 23F OPT in HIV-negative volunteers was significantly higher at 912± 59 compared to group A (255 ± 72, p≤0.001) and group B (439± 148, p≤0.001).

**Figure 3:**
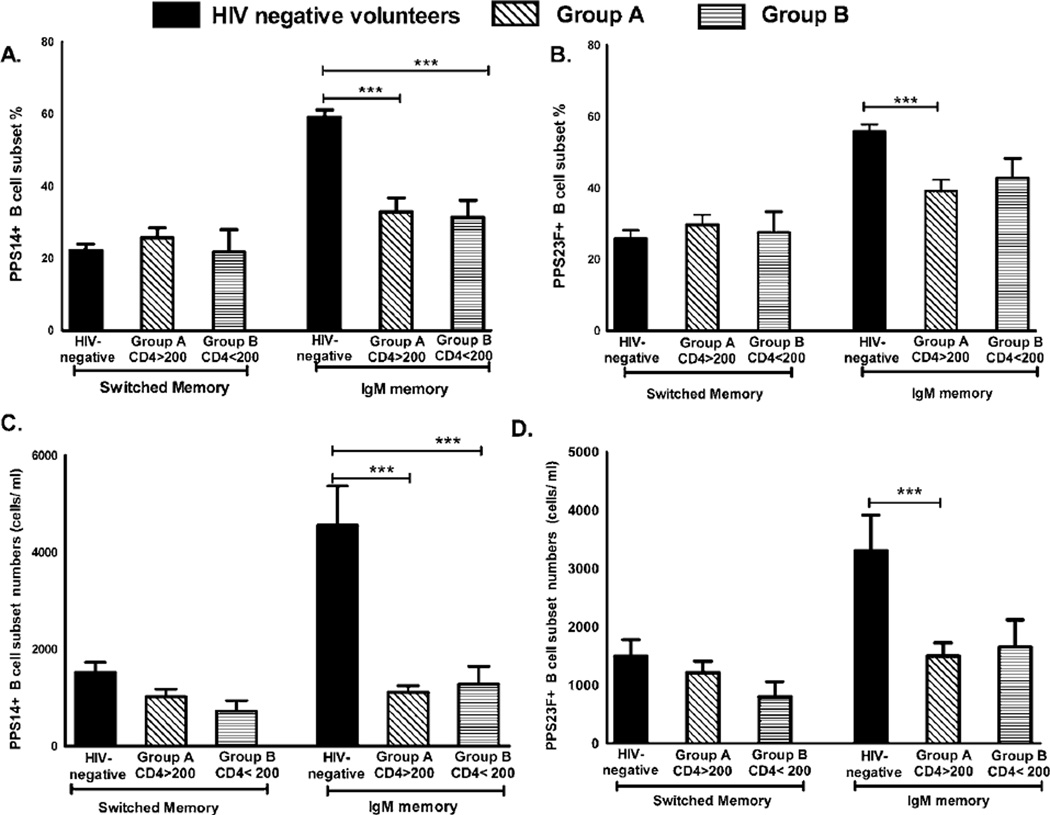
Comparative analysis of post-vaccination PPS-specific B cell response in long term HAART cohorts versus HIV negative volunteers *Panel A–B*: Post vaccination (Day 7) PPS14 (A) and 23F (B) -specific switched and IgM memory B cell percentages were compared between HIV-negative volunteers (n=22, black bars), group A (n=29, slanted stripes) and group B (n=10, horizontal stripes). Panel C–D: Post vaccination PPS14 (C) and 23F (D) -specific switched and IgM memory B cell numbers were compared between HIV-negative volunteers (n=22, black bars) compared to group A (n=29, slanted stripes) and group B (n=10, horizontal stripes).100,000 events were recorded for each sample. Group comparisons were performed using analysis of variance (ANOVA) with Tukeys post-hoc test. Data are represented as mean + standard errors of the mean (SEM). *p<0.05, **p<0.01, ***p<0.001.

We compared PPS-specific B cell phenotype in HIV-negative volunteers to the HAART cohorts. Consistent with our previous results [18], PPS-specific IgM memory B cells were the predominant cells to respond to PPV23 in HIV-negative volunteers. Although long term HAART cohorts showed a rise in the percentage of PPS-specific IgM memory B cells and absolute numbers on day 7 (Figure 2A–2D), both the percentage and absolute numbers of PPS-specific IgM memory B cells remained significantly lower in these groups compared to HIV-negative volunteers on day 7 post-vaccination (Figure 3). PPS-specific switched memory B cell percentage and numbers remained comparable between HIV-negative volunteers and group A and B (Figure 3).

Post-vaccination, PPS-14 specific IgM memory B cell percentage was significantly higher in HIV-negative volunteers at 59.05 ± 2.10 % compared to 32.84 ± 3.88 % (p ≤ 0.05) in group A and 31.34 ± 4.71 % (p ≤ 0.05) group B (Figure 3A). Similarly, PPS23F-specific IgM memory B cell percentage was higher at 55.90 ± 1.99% compared to 39.18 ± 3.12 % (p ≤ 0.05) in group A and 42.87 ± 5.43% (p ≤ 0.05) in group B (Figure 3B).

PPS14-specific IgM memory B cell numbers were significantly higher in HIV-negative volunteers at 4561 ± 805 cells/ml compared to 1109 ± 142 cells/ml in group A (p ≤ 0.05) and 1281 ± 368 cells/ml in group B (p ≤ 0.05) (Figure 3C). PPS23F-specific IgM memory B cell numbers were higher in HIV-negative volunteers at 3308 ± 611 cells/ml compared to 1458 ± 232 cells/ml in group A (p ≤ 0.05) and 1653 ± 467 cells/ml in group B (Figure 3D).

## Discussion

It is recommended that people at high risk for pneumococcal infection including the HIV-infected population, be revaccinated with PPV23 five years after primary vaccination. However, there is no data available to support this practice. The primary objective of the current study was to evaluate the immune response to PPV23 revaccination in long term HAART cohorts and to determine if this recommendation is beneficial as measured by surrogates of protection. Secondly, we sought to understand if the recovery in CD4 T cells post HAART also leads to a simultaneous improvement in antigen-specific B cell response to PPV23. This was then compared to the response in HIV-negative volunteers. Since revaccination is not a recommendation in healthy, HIV-negative volunteers, our study comprised of individuals who were recipients of primary PPV immunization. Although recent CDC guidelines recommends use of pneumococcal conjugate vaccine Prevnar (PCV13) followed by PPV23 for HIV-infected individuals [7], our study is important in the context of re-immunization in long term HAART cohorts as we determined the response at both the serological and antigen-specific B cell level.

We chose serotypes 14 and 23F for our study since they are included in PPV23 vaccine, have different charges on their surface and vary in immunogenicity [20,21]. Additionally, they formed the basis for comparison with our work on HIV-negative volunteers [18]. Our results indicate moderate to suboptimal change in IgG and IgM levels post vaccination against both PPS14 and 23F. Despite this, OPT against serotype 14 and 23F were significantly higher pre- to post-vaccination in both groups A and B. These discrepancies and poor correlation between antibody levels and OPT post PPV-vaccination are consistent with that in literature reinforcing the importance of including opsonophagocytic assays in studies [29]. Although correlates of protection have not been defined for adults, an OPT ≥8 is regarded as a threshold for response [26,27]. In our study, we noted significant pre- to post-vaccination rise in OPT in HAART cohorts exceeding the threshold value of 8. Our results suggest beneficial effects of PPV23 revaccination in long term HAART cohorts. Our results are in contrast to reports that suggest PPV23 induces hyporesponsiveness [30]. It must be noted that in these contrasting studies, conclusions were drawn solely on the basis of IgG levels [30]. We did however find significantly diminished serological response in groups A and B compared to HIV-negative volunteers.

Given the diminished serological response, we speculated there might be differences in the distribution of PPS-specific B cells between HIV-negative and long term HAART cohorts. Consistent with the speculation that IgM memory B cells play a critical role in response against *S.pneumoniae* [19,31], we identified PPS-specific B cells to be predominantly of the IgM memory phenotype in HIV-negative volunteers [18]. These cells play a pivotal role in protection against PPS, an effect that cannot be compensated by other PPS-specific B cell subsets as reported in our work in elderly, another high risk group for IPD [21].

In this study, we noted significantly lower percentages of PPS-specific IgM memory B cells in long term HAART cohorts despite PPV23 revaccination. Positive correlations between the percentages and numbers of PPS-specific IgM memory B cells (but not unselected or other PPS-specific subsets) with OPT strongly suggests the importance of these cells in protection against *S.pneumoniae*. Our findings are similar to reports where depletion of specific memory B cell pools during the early stages of HIV infection culminated in attenuated response to vaccines or were risk factors for pneumococcal infections [17,32].This finding is not surprising given that IgM memory B cells produce natural IgM which are efficient at clearing encapsulated organisms due to its superior complement fixing abilities [25,33].

We assessed the influence of CD4 T cells in serological response to PPS by comparing the response in group A (CD4>200) versus B (CD4<200). It should be noted that both groups A and B had a history of CD4 ≤ 200.We did not note significant differences in total and functional antibody response between the two groups despite differences in CD4 counts. Consistently, we did not note significant difference in PPS-specific IgM memory B cell percentage and numbers pre to post-vaccination between the 2 groups. Our results are consistent with other reports where CD4 T cells were suggested to be poor predictors in the vaccine response to PPV23 and with reports that suggest loss of discrete memory B cell subsets leads to heightened susceptibility to pneumococcal infections [9,10,34].

It has been reported that HIV can remain latent in the secondary lymphoid organs even in patients with undetectable plasma viral load despite administration of HAART [35,36]. It is thus plausible that HIV causes early damage to key B cell subsets that are required for response against PPS and these cells fail to recover with HAART. Our results are in alignment with other reports indicating HAART can only partially correct B cell associated perturbations [37].

To our knowledge, we are the first to elucidate the phenotype of PPS-specific B cells in long term HAART, PPV23 revaccinated volunteers. The specificity and sensitivity of direct PPS labeling was demonstrated in our previous work [18]. Furthermore, we assessed anti-PPS IgG and IgM response in these volunteers. Previous reports have focused on anti-PPS IgG undermining the relevance of IgM in protection [30,38]. Similarly, many studies failed to demonstrate functional antibody levels by OPA [30,38,39]. It is well accepted that OPTs are better indicators of protective immunity [27].

We thus conclude PPV23 revaccination to be beneficial for HIV-positive HAART experienced individuals. Comparable OPT between patient groups indicate poor PPS-specific B cell reconstitution irrespective of the degree of T cell reconstitution post-HAART. The response elicited by this population is however lower than the response seen in HIV-negative volunteers emphasizing the need for alternative approaches that can lead to a more robust response. Our findings emphasize the importance of evidence based vaccination practices for high risk individuals.

ACIP recently updated vaccine recommendations for HIV-positive individuals. Administration of 13-valent pneumococcal conjugate vaccine (PCV-13) followed by a dose of PPV23 is now recommended [7]. It remains to be seen if combined regimen can lead to improved protection against *S. pneumoniae* in long term HAART cohorts.

## Supplementary Material

Supplementary file-421

## Figures and Tables

**Table 1:** Baseline Characteristics of HIV-positive individuals recruited for the current study.

Parameters	Group A (CD4>200)	Group B (CD4<200)
n value	29	10
Age(years)	49	50
Sex, no. male/no. female	24/5	10/0
Race: White/Black/Hispanic/Asian	21/8/0/0	4/5/1/0
**HAART status**		
At the time of primary vaccination	No	No
At the time of re-vaccination (current study)	≥5 years	≥5 years
**Viral Load**		
At the time of revaccination (current study)	≤40 copies/ml	≤40 copies/ml
**PPV vaccination history**		
Primary Vaccination	Yes (≥5 years ago)	Yes (≥5 years ago)
Revaccinated (for the current study)	Yes	Yes
**CD4 count (cells/mm^3^)**		
Nadir CD4 history	≤200	≤200
CD4 count at the time of re-vaccination	≥200	≤200
**Total CD19+ B cells**¥ Percentage (%) Cells count (cells/µl)	8.5 ± 0.6 148±15	10.8 ± 1.4 151±33
**Unselected CD19+ B cell subset percentage (%)**¥		
1. Switched Memory (CD27+ IgM−) 2. IgM memory (CD27+ IgM+) 3. Naive (CD27−IgM +/−)	15.74 ± 1.68 9.23 ± 1.39 76.44 ± 2.48	12.79 ± 3.13 7.81 ± 1.51 79.39 ± 3.88
**Unselected CD19+ B cell subset count (cells/µl)** ¥ € 1.Switched Memory (CD27+ IgM−) 2.IgM memory (CD27+ IgM+) 3.Naive (CD27− IgM +/−)	23 ± 3 13 ± 2 114 ±14	20 ± 6 10 ± 3 122 ± 29

¥ Data are represented as mean ± standard error of the mean (SEM).

€ Cell counts of specific cell population in donor groups are reported as the average value of = [(percentage of specific cell population) * total lymphocytes/ml] for that group

**Table 2:** Serum anti-PPS antibody response post-PPV23 revaccination in long term HAART cohorts.

**A. anti-PPS IgG (µg/ml)**				
**Patient groups**	**PPS14** **Day 0**	**PPS14** **Day 30**	**PPS23F** **Day 0**	**PPS23F** **Day 30**
Group A (CD4>200)	5.24 ± 1.65	14.23 ± 3.16***	1.84 ± 0.80	4.74 ± 2.24
Group B (CD4<200)	5.48 ± 1.86	15.68 ± 4.12*	1.00 ± 0.64	1.94 ± 0.66
**B. anti-PPS IgM(µg/ml)**				
**Patient groups**	**PPS14** **Day 0**	**PPS14** **Day 30**	**PPS23F** **Day 0**	**PPS23F** **Day 30**
Group A (CD4>200)	0.37 ± 0.08	0.57 ± 0.15	0.16 ± 0.03	0.20 ± 0.04
Group B (CD4<200)	0.21 ± 0.05	0.28 ± 0.07	0.11 ± 0.02	0.14 ± 0.02
**C. Opsonophagocytic titers (OPT)**				
**Patient groups**	**Serotype 14** **Day 0**	**Serotype 14** **Day 30**	**Serotype 23F** **Day 0**	**Serotype 23F** **Day 30**
Group A (CD4>200)	3.0 ± 0.39	1412 ± 376 **	5 ± 1	255 ± 72 **
Group B (CD4<200)	5 ± 1	602 ± 435	6 ± 1	439± 148*

Data is represented as mean ± standard error of the mean.

* indicates significant rise in the post vaccination (day 30) antibody levels, µg/ml (panels A, B) or OPT (panel C) compared to pre-vaccination (day 0) within a group. Pre to post vaccination changes in a single group were compared using paired t-test and between groups were compared using analysis of co-variance (ANCOVA).

* p<0.05,

** p<0.01,

*** p<0.001.

**Table 3:** PPS-selected CD19+ B cell percentages (%) and absolute numbers (cells/ml) in long term HAART cohorts pre and 7 days post PPV23 revaccination.

**A.**				
**Patient groups**	**CD19^+^PPS14^+^** % **Day 0**	**CD19^+^PPS14^+^** % **Day 7**	**CD19^+^PPS23F^+^** % **Day 0**	**CD19^+^PPS23F^+^** % **Day 7**
Group A (CD4>200)	1.37 ± 0.29	2.87 ± 0.30***	1.29 ± 0.23	2.76 ± 0.23***
Group B (CD4<200)	0.69 ± 0.11	2.95 ± 0.74**	0.62 ± 0.11	2.22 ± 0.41**
**B.**				
**Patient groups**	**CD19^+^PPS14^+^** **(cells/ml)** **Day 0**	**CD19+PPS14+** **(cells/ml)** **Day 7**	**CD19+PPS23F+** **(cells/ml)** **Day 0**	**CD19+PPS23F+** **(cells/ml)** **Day 7**
Group A (CD4>200)	2006 ± 565	3696 ± 413**	1374 ± 202	3809 ± 441***
Group B (CD4<200)	1036 ± 280	3768 ± 758**	1038 ± 280	3330 ± 628**

Data are mean ± standard error of the mean.

* indicates significant rise in the post vaccination (day 7) levels compared to pre-vaccination (day 0) within a group. Cell counts of specific cell population in donor groups are reported as the average value of = [(percentage of specific cell population) * total lymphocytes/ml] for that group. Pre to post vaccination changes in a single group were compared using paired t-test and between groups were compared using analysis of co-variance (ANCOVA).

* p<0.05,

** p<0.01,

*** p<0.001.

**Table 4:** Unselected (PPS−) versus PPS-selected (PPS+) CD19+ B cell subset percentages (%) in long term HAART cohort’s 7 days post PPV23 vaccination.

**A. Switched Memory B cell subset distribution (%)**				
**Patient groups**	**PPS14^−^ (Unselected)**	**PPS14^+^ (Selected)**	**PPS23F^−^ (Unselected)**	**PPS23F^+^ (Selected)**
Group A (CD4>200)	15.7 ± 1.6	25.69 ± 2.75***	15.74 ±1.68	29.62 ± 2.89***
Group B (CD4<200)	12.76 ± 2.81	21.78 ± 6.19	12.79 ± 3.13	27.59 ± 5.77*
**B. IgM Memory B cell subset distribution**				
**Patient groups**	**PPS14^−^ (Unselected)**	**PPS14^+^ (Selected)**	**PPS23F^−^ (Unselected)**	**PPS23F^+^ (Selected)**
Group A (CD4>200)	9.30 ± 1.39	32.84 ± 3.88***	9.23 ± 1.36	39.18 ± 3.12***
Group B (CD4<200)	8.16 ± 1.79	31.34 ± 4.71**	7.81 ± 1.51	42.87 ± 5.43***
**C. Naive B cell subset distribution**				
**Patient groups**	**PPS14^−^ (Unselected)**	**PPS14^+^ (Selected)**	**PPS23F^−^ (Unselected)**	**PPS23F^+^ (Selected)**
Group A (CD4>200)	74.98 ± 2.38	41.46 ± 3.56***	76.24 ± 2.48	31.25 ± 2.66***
Group B (CD4<200)	79.0 ± 3.81	46.87±6.34***	79.39 ± 3.88	26.23 ± 6.97***

Data are mean ± standard error of the mean.

* indicates significant difference in the PPS-selected population post vaccination (day 7) levels compared to unselected B cells within a group. Pre to post vaccination changes in a single group were compared using paired t-test and between groups were compared using ANCOVA.

* p<0.05,

** p<0.01,

*** p<0.001.

**Table 5:** Unselected (PPS−) versus PPS-selected (PPS+) CD19+ B cell subset numbers (cells/ml) in long term HAART cohort’s pre and 7 days post PPV23 revaccination.

**A. Switched Memory B cell subset distribution (cells/ml)**				
**Patient groups**	**PPS14^−^ (Unselected)** **Day 0**	**PPS14^+^ (Selected)** **Day 7**	**PPS23F^−^ (Unselected)** **Day 0**	**PPS23F^+^ (Selected)** **Day 7**
Group A (CD4>200)	624 ± 200	1022 ± 155	525 ± 104	1215 ± 196**
Group B (CD4<200)	159 ± 48	731 ± 212*	162 ± 28	1653 ± 467*
**B. IgM Memory B cell subset distribution (cells/ml) (%)**				
**Patient groups**	**PPS14^−^ (Unselected)** **Day 0**	**PPS14^+^ (Selected)** **Day 7**	**PPS23F^−^ (Unselected)** **Day 0**	**PPS23F^+^ (Selected)** **Day 7**
Group A (CD4>200)	317 ± 60	1109 ± 142***	331 ± 47	1458 ± 233***
Group B (CD4<200)	184 ± 56	1281 ± 368*	239 ± 64	1653 ± 467**
**C. Naive B cell subset distribution (cells/ml) (%)**				
**Patient groups**	**PPS14^−^ (Unselected)** **Day 0**	**PPS14^+^ (Selected)** **Day 7**	**PPS23F^−^ (Unselected)** **Day 0**	**PPS23F^+^ (Selected)** **Day 7**
Group A (CD4>200)	1116 ± 326	1503 ± 235	524 ± 101	1212 ± 158***
Group B (CD4<200)	675 ± 220	555 ± 135	635 ± 205	907 ± 250

Data are mean ± standard error of the mean.

* indicates significant rise in the post vaccination (day 7) levels compared to pre-vaccination (day 0) within a group. Cell counts of specific cell population in donor groups are reported as the average value of = [(percentage of specific cell population) * total lymphocytes/ml] for that group. Pre to post vaccination changes in a single group were compared using paired t-test and between groups were compared using analysis of co-variance (ANCOVA).

* p<0.05,

** p<0.01,

*** p<0.001.
